# Personal News Items

**Published:** 1915-01

**Authors:** 


### Personal News Items.

Dr. Roscoe Bristow, of Amarillo, visited friends in Illinois during the holidays.

Dr. J. L. Daily and family, of San Saba, spent Xmas with relatives at Cherokee.

Dr. R. W. Wallis, of Rockdale, spent the holidays in his old home at Arkadelphia, Ark.

Dr. J. E. Nevill, of Bonham, attended a meeting of the North Texas Medical Association.

Dr. and Mrs. J. W. Tollison are receiving congratulations over the advent of a 10-pound son.

Dr. Sweatland, of Nacogdoches, was called to Houston December 9th on professional business.

Dr. L. S. Payne, of Troy, recently had the misfortune of having both his hands badly burned.

Dr. R. A. Nicholson, of Devine, has been confined to his home with a bad case of sore eyes this week.

Dr. Fyke authorizes the announcement of Dr. W. A. Davis, of Jourdanton, as Registrar of Vital Statistics.

Dr. Ross May and Dr. C. P. Johnson, of Whitewright, attended the North Texas Medical Association at Dallas.

Drs. Fiske and Parks, of Lancaster, attended the meeting of the North Texas Medical Association in Dallas.

Dr. J. J. Adkins, of Refugio, is seriously ill.

Dr. Deal, of Nacogdoches, was operated on for appendicitis on the 9th of December. He is getting along nicely.

Dr. and Mrs. J. E. Nunn, of Amarillo, have sailed from Galveston on a trip to Havana, Cuba, and other points.

Dr. Daune Meredith and Everett Jones, of Wichita Falls, were the guests of the North Texas Medical Association.

Dr. C. R. Hartsook, of Wichita Falls, has returned from New York, where he has been doing post-graduate work.

Dr. Frank Seay, of Georgetown, Texas, has returned from the Temple Sanitarium, and is rapidly regaining his strength.

Dr. T. S. Edwards, wife and little son, of Knox City, Texas, spent the holidays with relatives in Blanco and San Marcos.

Dr. Oscar Kruger, of Caldewll, Texas, has returned from Vienna, Austria, where he has been doing post-graduate work.

Dr. B. S. Halliburton, of Devine, is confined to his room and bed with a complication of troubles that show little improvement.

Dr. and Mrs. R. M. Payne and children, of Sulphur Springs, spent Christmas in Weaver visiting the doctor's mother, Mrs. M. C. Payne.

Dr. Singleton, of Dalhart, has bought an interest in the Norwood Osteopathic Sanitarium at Mineral Wells, and will move to that city.

Dr. N. Shilling, of Cedar Bayou, Texas, was elected to honorary fellowship in the Southern Surgical and Gynecological Association.

Dr. Chas. Carter, who has been practicing his profession near Omega, has a position in the T. C. U. Medical Department at Fort Worth.

Dr. and Mrs. G. L. Shely, of Valley Mills, were made happy by the arrival of a pretty girl baby at their home Monday, November 7th.

Dr. and Mrs. J. H. Combs, of San Marcos, recently celebrated their fiftieth anniversary. The occasion was most pleasant for all who attended.

Dr. C. C. Jones, of Comfort, Texas, who left two months ago for Chicago and other Northern cities to study new surgical work, has returned.

Dr. John Preston, Superintendent of the Insane Asylum at Austin, who has been quite ill, is improving, much to the delight of his many friends.

Drs. S. L. and O. N. Mayo, of Belton, will in the near future open a private hospital, which will be well fitted up with the latest modern hospital equipment.

Dr. and Mrs. C. W. Simpson, of Waxahachie, left for Boston December 20th to spend the holidays with their son, Charles, who is attending Harvard University.

Dr. John W. Burns, of Cuero, reached home after a stay of several weeks in New York and Rochester, Minn., where he attended lectures and took special surgical courses.

At the recent session in Washington the American College of Surgeons gave the degree of fellowship to several of the eminent surgeons of Texas, among them being Dr. Frank L. Barnes, of Trinity.

Dr. H. G. Thomas, a prominent physician of Handley, is critically ill at his home. He is said to be suffering from traumatic pneumonia. His chest was injured two years ago in an accident, and it is still giving him trouble.

Dr. E. L. Gilcreest, of Gainesville, Texas, is being strongly solicited by the National Red Cross Association to go to Europe and engage in the hospital Red Cross work as surgeon.

Dr. O. B. Atkinson, of Florence, has been appointed Deputy Grand Master of Williamson county by Hon. Jewel P. Lightfoot, Grand Master of the Grand Lodge of Masons of Texas.

Drs. E. L. Gilcrest, J. E. Gilcrest, E. M. Parrish, R. C. Whiddon, O. R. Johnson, C. F. Rice and J. C. Jennette, of Gainesville, attended the meeting of North Texas Medical Association.

Bob Hoffman, newly-appointed Dairy and Pure Food Commissioner, announced the following inspectors: Dr. W. H. Minton, Fort Bend; Richard Hudson, Collin; Mrs. C. L. Darwin, Cooke county, and R. A. Malone, Travis county.

Dr. and Mrs. Meeks, of Hemphill, had a very narrow escape when their car turned a complete circle, rolled entirely over and headed in the opposite direction. The doctor was glad to escape with no less injury than three broken ribs.

Dr. H. L. Moore, of Dallas, was again elected Secretary of the North Texas Medical Association at the meeting of that body in Dallas. Dr. Moore has been re-elected to this position from time to time for the past twelve or fourteen years.

Dr. William Brumby, President of the San Antonio Board of Health, has tendered his resignation from that body, and on January 1st will move with his family to Waco to become chief surgeon for the Amicable Life Insurance Company.

Dr. E. H. Cary entertained the members of the Dallas Medical and Surgical Society in his home on the evening of December 6. The early hours of the evening were devoted to papers and discussion, while the latter part was given over to social pleasures.

Dr. Frank Clark, Sr., and son, Joe Frank, of Iowa Park, Texas, attended the Wichita County Medical Association at Wichita Falls, where Dr. Clark, Sr., read a paper on pneumonia. Dr. Joe Frank was a delegate to the Panhandle Convention at Memphis.

Dr. W. F. Thackery, of Fowlerton, has started a new industry in the way of raising castor beans. He has threshed several tons, and yet has a considerable quantity that are to be harvested. The doctor is of the opinion that growing these beans can be made a profitable industry in Southwest Texas.

Dr. R. W. Knox, of Houston, at the recent organization of the American College of Surgeons, was elected a charter member of that organization. We congratulate the association upon its good taste and discernment. Dr. Knox is one of the really big men of Texas, though he doesn't make much noise about it.

Dr. M. M. Carr, formerly interne at the city hospital of Dallas, has been appointed by Dr. A. W. Nash City Health Officer, to succeed Dr. Charles Card as house physician and steward of that hospital. Dr. Card resigned to enter private practice. He has been connected with the hospital for a number of years.

The announcement of the following appointments will doubtless prove interesting to the physicians of Texas:

State Health Officer, Dr. Ed. Fyke, Fort Worth.

State Board of Health, Dr. L. W. Hollis, Abilene; Dr. C. W. Hoeflich, Houston; Dr. L. M. Weinfield, San Antonio; Dr. Hugh L. McLaurin, Dallas; Dr. W. D. Littler, Fort Worth; Dr. H. J. Childress, Gilmer; Dr. E. M. Wood, Georgetown.

Dr. Fyke, after conferring with Governor-elect Ferguson, announced the following appointments as quarantine officers: Dr. E. S. McCain, Brownsville; Dr. M. L. D. Jordan, Velasco; Dr. E. O. Arnold, Aransas Pass; Dr. Justus S. Davidson, Galveston; Dr. H. C. Hall, Laredo.

